# Interconnections Between RNA-Processing Pathways Revealed by a Sequencing-Based Genetic Screen for Pre-mRNA Splicing Mutants in Fission Yeast

**DOI:** 10.1534/g3.116.027508

**Published:** 2016-03-25

**Authors:** Amy Larson, Benjamin Jung Fair, Jeffrey A. Pleiss

**Affiliations:** Department of Molecular Biology and Genetics, Cornell University, Ithaca, New York 14853

**Keywords:** *Schizosaccharomyces pombe*, genetic screen, genomics, heterochromatin, pre-mRNA splicing

## Abstract

Pre-mRNA splicing is an essential component of eukaryotic gene expression and is highly conserved from unicellular yeasts to humans. Here, we present the development and implementation of a sequencing-based reverse genetic screen designed to identify nonessential genes that impact pre-mRNA splicing in the fission yeast *Schizosaccharomyces pombe*, an organism that shares many of the complex features of splicing in higher eukaryotes. Using a custom-designed barcoding scheme, we simultaneously queried ∼3000 mutant strains for their impact on the splicing efficiency of two endogenous pre-mRNAs. A total of 61 nonessential genes were identified whose deletions resulted in defects in pre-mRNA splicing; enriched among these were factors encoding known or predicted components of the spliceosome. Included among the candidates identified here are genes with well-characterized roles in other RNA-processing pathways, including heterochromatic silencing and 3ʹ end processing. Splicing-sensitive microarrays confirm broad splicing defects for many of these factors, revealing novel functional connections between these pathways.

The protein-coding regions of most eukaryotic genes are interrupted by noncoding introns, which must be precisely removed from the pre-mRNA in order to generate a translatable message. This essential process is carried out by the spliceosome, a dynamic macromolecular machine that recognizes specific sequence elements within the pre-mRNA, such as the short consensus sequences that mark intron boundaries, and catalyzes intron removal (Will and Lührmann 2011). At its core, the spliceosome is composed of five snRNA-protein complexes (snRNPs), each comprised of a single RNA and multiple core protein factors. In addition, the human spliceosome associates with upwards of 200 auxiliary splicing proteins that aid in proper recognition of splice sites and catalysis (Wahl *et al.* 2009).

Over the last decade, it has become increasingly clear that splicing is integrated with other steps of pre-mRNA synthesis and maturation. Studies from yeast to humans suggest that the majority of splicing occurs cotranscriptionally while the RNA is still tethered to the polymerase (Core and Lis 2008; Pandya-Jones and Black 2009; Carrillo Oesterreich *et al.* 2010; Ameur *et al.* 2011; Khodor *et al.* 2011). Accordingly, multiple lines of evidence support the idea that transcriptional dynamics influence the splicing process. Mutations that alter polymerase elongation rate yield different splicing patterns (de la Mata *et al.* 2003; Dujardin *et al.* 2014), suggesting a kinetic coupling between transcription rate and the ability of splicing factors to recognize and act upon splice sites. Genome-wide studies have also demonstrated that transcriptional pausing coincides with the splicing process (Core and Lis 2008; Alexander *et al.* 2010). In addition to a kinetic coupling of elongation and splicing, biochemical studies have shown that the C-terminal domain (CTD) of RNA polymerase II can directly interact with splicing components (Vincent *et al.* 1996; Misteli and Spector 1999), and that posttranslational modifications of the CTD can differentially impact recruitment of splicing components (Morris and Greenleaf 2000; Muñoz *et al.* 2010; David and Manley 2011). The chromatin environment encountered by the transcribing polymerase can also influence splicing, and genome-wide nucleosome positioning data from fission yeast to humans reveal an enrichment of nucleosome density in exons over introns (Schones *et al.* 2008; Andersson *et al.* 2009; Spies *et al.* 2009; Tilgner *et al.* 2009; Wilhelm *et al.* 2011; Iannone *et al.* 2015). The mechanistic link between chromatin state and splicing could be explained, in part, by the relation between chromatin state and polymerase kinetics (Li *et al.* 2007; Luco *et al.* 2011), but might also reflect direct interactions between chromatin marks and splicing factors. For example, the H3K4me3 mark interacts with the U2 snRNP through interactions with the adapter protein CHD1 (Sims *et al.* 2007). In addition to chromatin-based interactions, it is clear that the cleavage and polyadenylation machinery at the 3ʹ end of transcripts can interact with splicing components to influence splicing. In higher eukaryotes, identification of splice sites in terminal introns requires interactions between splicing components and the cleavage and polyadenylation machinery (Berget 1995). Recently, the cleavage and polyadenylation factor CPSF1 was found to regulate alternative splicing in human T-cells (Evsyukova *et al.* 2013). These interconnections between splicing and other nuclear processes underscore the need for unbiased genome-wide approaches to identify the full complement of factors that functionally impact spliceosomal activity.

The fission yeast, *Schizosaccharomyces pombe*, provides a powerful genetic system in which to examine the splicing pathway. Like the budding yeast, *Saccharomyces cerevisiae*, fission yeast is genetically tractable, allowing for easy manipulation of its genome. The *S. cerevisiae* genome, however, has shed most of its introns, with only ∼300 introns remaining (Kupfer *et al.* 2004). In contrast, over 5000 introns have been identified in the *S. pombe* genome, and over 1000 genes are interrupted by multiple introns (Wilhelm *et al.* 2008; Rhind *et al.* 2011). Furthermore, whereas splice site sequences in budding yeast introns tend to conform to a strict consensus sequence, *S. pombe* splice sites are characterized by a far higher level of degeneracy (Kupfer *et al.* 2004), more closely resembling the degeneracy seen in human splice sites. Perhaps accordingly, sequence homology identifies many auxiliary components of the human spliceosome, such as SR proteins, that are present in the *S. pombe* genome but have been lost in the *S. cerevisae* lineage (Käufer and Potashkin 2000; Webb *et al.* 2005). These properties suggest that regulation of pre-mRNA splicing in *S. pombe* may be more similar to that seen in humans than in *S. cerevisiae* (Ram and Ast 2007). Indeed, some transcripts in *S. pombe* are subject to mammalian-like, environmentally regulated exon skipping (Awan *et al.* 2013), and others respond to insertion of mammalian splicing enhancer elements (Webb *et al.* 2005). Moreover, similar to observations in mammalian cells, widespread activation of cryptic splice sites has been demonstrated in *S. pombe*, highlighting the flexibility in the *S. pombe* spliceosome for selecting splice sites (Bitton *et al.* 2015; Stepankiw *et al.* 2015). Although these features highlight the potential of *S. pombe* to serve as a model for understanding the complex splicing seen in higher eukaryotes, the precise factors responsible for regulating these splicing events remain largely unknown.

Components of the *S. pombe* spliceosome have been identified using a variety of approaches. Genetic screening of randomly mutagenized strains identified numerous core splicing factors (Potashkin *et al.* 1989; Rosenberg *et al.* 1991; Alahari *et al.* 1993; Habara *et al.* 1998), and biochemical purifications followed by mass spectrometry have greatly added to the list of components (Ohi *et al.* 2002; Chen *et al.* 2014). Although these strategies successfully identified core components of the spliceosome, they have been less effective at identifying factors that functionally connect splicing with other nuclear processes. More recently, a high-throughput genetic interaction mapping strategy examining nonessential *S. pombe* genes identified strong genetic interactions between U2 snRNP components of the spliceosome and chromatin remodeling enzymes, such as the SWI/SNF complex (Patrick *et al.* 2015), suggesting that a mechanistic coupling between chromatin modification and splicing also exists in *S. pombe*. In addition, recent systematic genome-wide yeast-two-hybrid interaction mapping strategies have correctly identified a handful of *S. pombe* genes as factors in the splicing pathway, based on physical interactions with known spliceosome components (Ren *et al.* 2011; Vo *et al.* 2016). These high-throughput genetic and physical interaction strategies can yield a wealth of information and strongly hint at a gene’s involvement in the splicing pathway, but they do not provide a direct functional test of a factor’s impact on splicing.

We have previously described a reverse-genetic screening methodology in *S. cerevisiae* that couples high-throughput sample processing with quantitative RT-PCR to enable direct measurements of the splicing efficiency of endogenous pre-mRNA transcripts in the background of thousands of mutant strains (Albulescu *et al.* 2012). In addition to identifying the majority of known splicing mutants, this work successfully identified splicing defects associated with components of the SWI/SNF complex, as well as with components of the cleavage and polyadenylation machinery, confirming both the sensitivity of this approach and the evolutionarily conserved nature of these functional interactions. Here, we present the results of a study designed to identify nonessential genes in the *S. pombe* genome whose deletion impacts the splicing efficiency of endogenous transcripts. We have developed and implemented a sequencing-based approach for monitoring splicing efficiency in the background of thousands of *S. pombe* strains, and describe the functional significance of those mutants identified.

## Materials and Methods

### Strains and cell growth

All strains examined here were from the haploid deletion library from Bioneer (Kim *et al.* 2010), representing 3020 individual gene deletions, a complete list of which is available in Supplemental Material, Table S1. All strains were grown in supplemented rich growth medium (YES) at 32°, according to standard procedures (Forsburg and Rhind 2006), unless otherwise noted. Strains were recovered from glycerol stocks on solid media supplemented with 200 µg/ml G418. A manual pinning tool (V&P Scientific, cat.#: VP384FP6) was used to transfer cells from solid media into 384-well microtiter plates (Greiner BioOne, cat.#: 781271) for growth in liquid media. Liquid cultures were grown in an Infors HT Multitron plate shaker at 900 rpm with 80% constant humidity. Breathable adhesive tape (VWR, cat.#: 60941-086) was used to seal the plates and reduce evaporation. Because the growth rates of the strains being used varied substantially, an approach was developed to enable the collection of a similar number of actively growing cells for every strain. Initial cultures (150 µl) of all strains were grown in microtiter plates for 2 d, allowing nearly all strains to reach saturation. The cell density for most strains is similar at saturation, allowing us to effectively ‘normalize’ cell numbers. Using a liquid handling robot (Biomek NX), 2 µl of saturated culture was used to inoculate 150 µl of fresh media in duplicate to create biological replicate cultures for each strain. After inoculation, cells were allowed to grow for 8 hr, allowing most strains to reach A_600_ values near 0.5. Cells were harvested by centrifugation at 5000 × *g* for 5 min, and pellets were flash frozen in liquid N_2_ and stored at –80° until further processing.

### cDNA synthesis and library preparation

**Figure 1:**
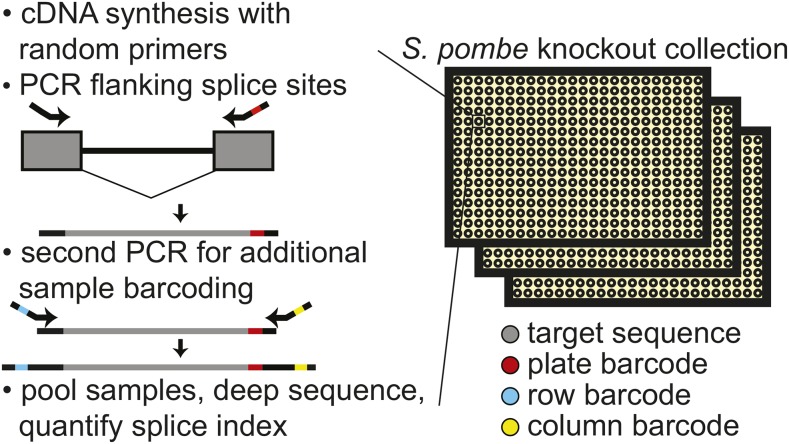
Schematic of workflow for quantitatively measuring splicing in the fission yeast deletion collection. After cell growth, RNA isolation, and cDNA synthesis with random primers, consecutive PCR reactions are performed using primers that flank an intron to amplify both spliced and unspliced RNA while appending sample specific barcodes and Illumina compatible ends. Estimates of splicing efficiency in each strain are determined by counting the number of spliced and unspliced sequencing reads derived from each sample. cDNA, complementary DNA; PCR, polymerase chain reaction.

RNA was isolated from cell pellets and cDNA was synthesized using random ninemers for primers, as previously described (Albulescu *et al.* 2012). The resulting cDNA was amplified by two sequential PCR reactions to generate products compatible with the Illumina HiSeq2000 Flow Cell as follows. For each cDNA sample, a 12 µl PCR reaction was prepared containing 1 × Phusion HF buffer (New England Biolabs), 1 × Phusion enzyme, 250 nM forward primer with custom plate-specific barcodes, 250 nM reverse primer, and 1% of the cDNA reaction. The plate-specific barcode sequences were designed as previously described (Mamanova *et al.* 2010). A complete list of the primers used in this study is available in Table S2. Cycling conditions for this first PCR reaction were as follows: 95**°** for 3 min, then an empirically determined number of cycles of 98**°** for 15 sec, 62**°** for 20 sec, and 72° for 30 sec. The number of amplification cycles required was determined in a separate QPCR reaction as the minimum number of cycles necessary to generate a detectable fluorescence signal; required cycle numbers varied from 18–21 for the different primers and plates. The products resulting from this first PCR contained plate-specific barcodes, but no well-specific barcodes (see Figure 1). For each target, the products from each plate of this first PCR reaction were pooled into a single 384-well microtiter plate, and 0.5 µl was used to seed a second PCR reaction (15 µl), during which well-specific Illumina-Nextera barcodes and Illumina Flow Cell binding sites were appended. This reaction contained 1 × Phusion HF buffer (New England Biolabs), 1 × Phusion enzyme, 200 nM forward Nextera index primer, and 200 nM Nextera reverse index primer. Cycling conditions were as follows: 95**°** for 3 min, then 5 cycles of 98**°** for 15 sec and 68**°** for 60 sec. The PCR products were pooled, concentrated via ethanol precipitation, purified using glass fiber spin columns (Zymo Research), and separated on a 6% native acrylamide gel. Materials of the expected molecular weight ranges were excised from the gel and recovered by soaking crushed gel bits in 0.3 M sodium acetate followed by ethanol precipitation. The resulting DNA precipitate was dissolved in 25 µl water and sequenced on the Illumina HiSeq2000 with the assistance of the Cornell University Biotechnology Resource Center.

### Data processing

Reads were demultiplexed using a combination of Nextera-specific indices and custom plate-specific barcodes (Mamanova *et al.* 2010) within the insert read. The bwa-mem aligner (Li and Durbin 2009) was then used to align reads to a custom index containing both the spliced and unspliced isoforms of the two targets. A splice-index (SI) was calculated for *fet5*_intron1 and *pwi1*_intron2 in each sample by comparing the number of reads mapped to the unspliced isoform *vs.* the spliced isoform as follows:

$$SI=\frac{unspliced\;read\;count}{spliced\;read\;count}$$

To determine SI relative to wild type while accounting for plate to plate variation, we assumed that the median SI within each 384-well plate was representative of wild type. Therefore, the relative SI was calculated as:

$$SI_{relative}=\frac{SI}{SI_{PlateMedian}}$$

**Figure 2:**
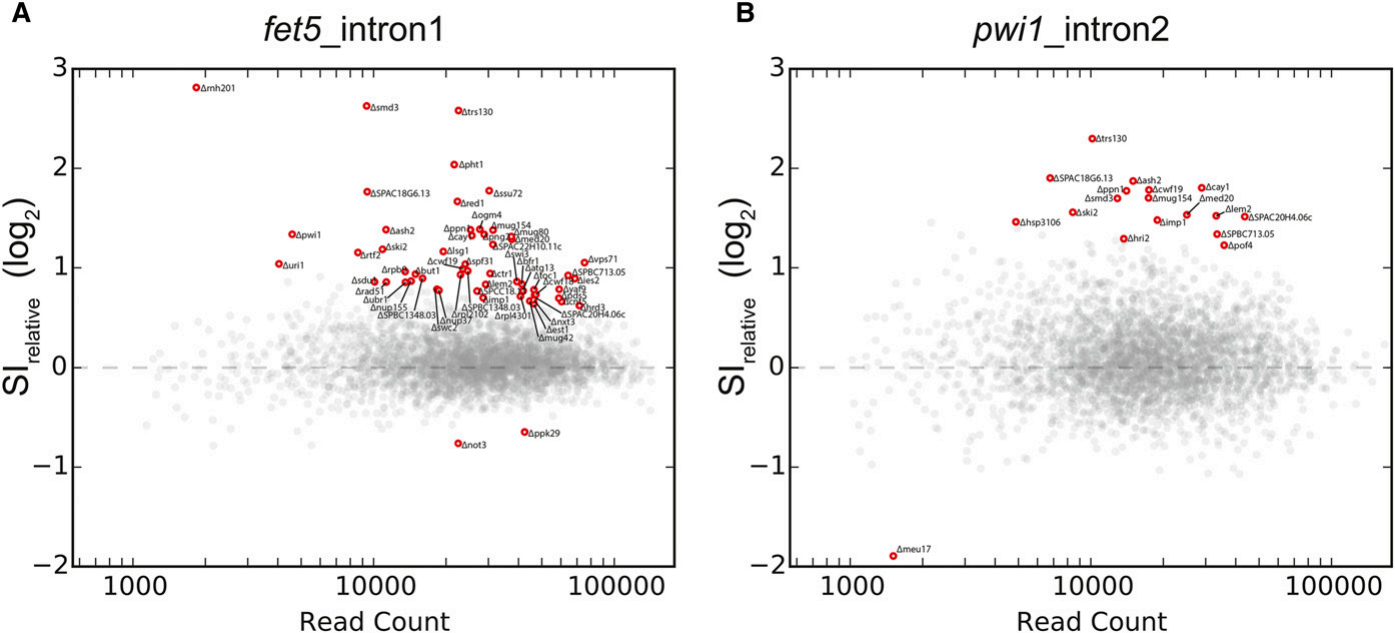
Relative splice index measurements in deletion strains. The relative splice index for *fet5*_intron1 (A) or *pwi1*_intron2 (B) is plotted as a function of read count for each of the ∼3000 strains examined. Strains that significantly differed from wild type after multiple hypothesis correction are colored red and labeled. A total of 61 strains were identified as having a significantly different splice index measurement for either *fet5*_intron1 or *pwi1*_intron2.

After determining the SI for each biological replicate, we filtered our dataset to include only those samples for which the standard deviation between the *log_2_(SI_relative_)* was less than 1, and for which the combined read count was greater than 1000. A total of 3007 and 3005 strains (99.6% and 99.5%) passed these quality scores for the *fet5*_intron1 and *pwi1*_intron2 datasets, respectively. In order to determine the subset of strains which exhibited a *log_2_(SI_relative_)* that was statistically significantly different from wild type, we considered how the precision of our *SI_relative_* measurements varied as a function of read count. In concept, this approach has similarities to algorithms commonly used for RNA-seq analysis, which empirically estimate noise within a dataset to identify significant changes in gene-expression (Love *et al.* 2014). The log_2_-transformed *SI_relative_* values were plotted as a function of log-transformed read count for each sample (see Figure 2). The dataset was then divided into 20 equal sized bins based on read count. Using the mean and standard deviation within the bins as data points, spline interpolation was used to estimate the log_2_-transformed mean (${µ}_{interpolated}$) and standard deviation ($\sigma_{interpolated}$) of SI_rel_ measurements at any given read count under the null hypothesis. For each strain, a *P*-value was then estimated by defining a Z-score as follows:

$$Z=\;\frac{\log_{2}\left(SI_{relative}\right)-{µ}_{interpolated}}{\sigma_{interpolated}}$$

**Figure 3:**
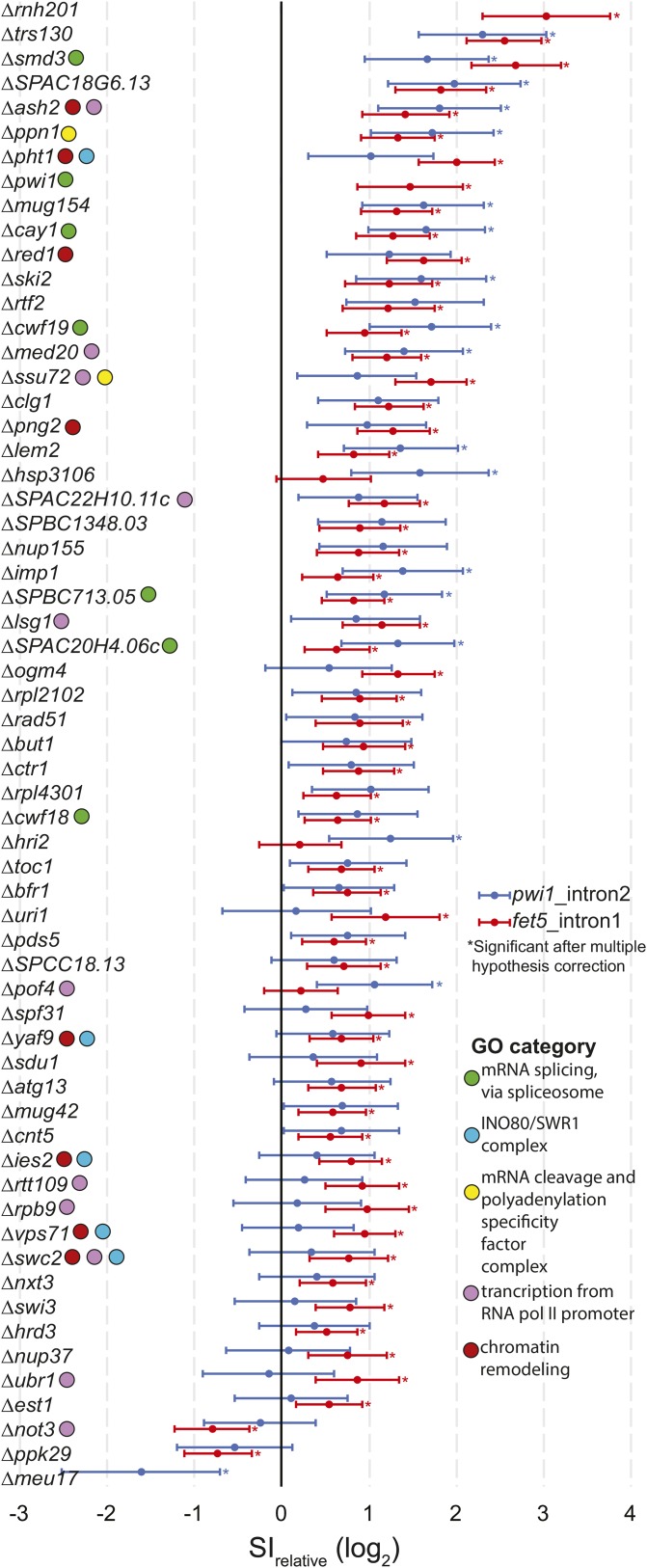
Gene deletions that result in significant splice index changes in either *fet5*_intron1 or *pwi1*_intron2. The measured relative splice index is shown for *fet5*_intron1 and *pwi1*_intron2, with 95% confidence intervals for the 61 gene deletion strains that were significantly different than wild type for at least one of the splicing events examined. Notable Gene Ontology (GO) categories are indicated. mRNA, messenger RNA; RNA pol II, RNA polymerase II; SI, splice-index.

Strains were called as significant if the Benjamini-Hochberg corrected *P*-value was below 0.05. The 95% confidence intervals in Figure 3 represent *log_2_(SI_relative_)* ± *2σ_interpolated_* given the read depth of that strain. The complete set of raw read counts and processed data for each strain are available in Table S1.

### Splicing sensitive microarrays

All microarrays were performed as two-color arrays comparing mutant and wild-type strains, each grown under identical conditions. Briefly, strains were grown to saturation at 30**°**, then back-diluted in 25 ml cultures and allowed to grow at 30**°** until they reached an optical density of A_600_ ∼0.5. Total cellular RNA samples were isolated, converted into cDNA, fluorescently labeled, and hybridized to the array as previously described (Inada and Pleiss 2010). Biological replicate microarrays were performed for most mutant strains, with average expression measurements between biological replicates being presented in the figures. Both raw and processed microarray data are available through GEO using accession number GSE79153.

### Data availability

The authors state that all data necessary for confirming the conclusions presented in the article are represented fully within the article.

## Results and Discussion

Here, we sought to identify the full complement of nonessential genes that impact pre-mRNA splicing efficiency in *S. pombe*, an organism whose splicing properties closely resemble those seen in higher eukaryotes (Ram and Ast 2007). To quantitatively measure the impact of mutations on the splicing pathway, we designed an assay that would allow for high-sensitivity detection of both spliced and unspliced isoforms in thousands of unique samples (see Figure 1). Briefly, cDNA from a given sample was used as template for a PCR reaction using primers that flank a splicing event, enabling amplification of both spliced and unspliced isoforms. By appending appropriately barcoded sequences, the resulting material was subjected to deep sequencing to count the number of molecules corresponding to both the spliced and unspliced isoforms for each sample. To demonstrate that this approach could provide a quantitative representation of the underlying species, we measured isoform ratios for samples that contained known ratios of different spliced isoforms. Across a large range of relative isoform abundances, this sequencing-based approach gave results that were both highly accurate and precise (Figure S1).

After determining that this sequencing approach was sensitive and quantitative, we turned to examining each of the ∼3000 deletion strains available within the *S. pombe* haploid deletion collection (Kim *et al.* 2010) to identify novel factors whose disruption impacts splicing. Primers were designed that would allow for the determination of the splicing efficiency of two introns: the single intron in the *fet5* transcript, a predicted GTPase involved in RNA polymerase localization, and the second intron in the *pwi1* transcript, a splicing coactivator. The *fet5* intron resembles a typical intron in *S. pombe*, in that the *fet5* transcript contains just a single intron with canonical 5′ splice site (GUAAGU), and branch point (UGCUAAU) sequences, and whose length [45 nt (nucleotides)] is close to the median intron length (56 nt). The second intron in *pwi1* is also of typical length (59 nt) for an *S. pombe* intron, and has a typical branch point sequence (CAUUAAU) but an atypical 5ʹ splice site sequence (GUACAA) which significantly deviates from the canonical sequence. Importantly, because these two introns are short, the PCR amplification efficiency of both the spliced and unspliced isoforms should be similar, reducing the likelihood of artifacts derived from amplification bias. In total, ∼12,000 samples were generated, corresponding to each of these targets within each of these strains with biological replicates. As a convenient measure of splicing efficiency, we define the splicing index (SI) as the ratio of unspliced to spliced reads, and looked to identify mutants that caused significant changes to the SI. Importantly, because this assay measures the steady state abundances of specific RNA species, a high SI could indicate a defect in pre-mRNA splicing, or alternatively, a change in the relative stabilities of spliced or unspliced RNA. For both the *fet5*_intron1 and *pwi1_*intron2, the unspliced pre-mRNA was present at about 2% of the spliced mRNA in the background of most strains, with respective median SI values of 0.018 and 0.025 (Figure S2, A and B), consistent with the expectation that splicing occurs efficiently and the vast majority of these transcripts are present as the spliced isoform. Moreover, for the vast majority of strains, the measured SI was relatively close to the median value, with interquartile ranges across all samples of 0.004 and 0.011 for the *fet5*_intron1 and *pwi1*_intron2 targets, respectively (Figure S2, A and B), consistent with the expectation that most genes examined here do not impact the splicing pathway. Across all strains, the biological replicates were correlated with R^2^ values of 0.37 and 0.23 for *fet5*_intron1 and *pwi1*_intron2, respectively (Figure S2, C and D).

As with any RNA-sequencing experiment, the statistical power to identify changes in expression increases with greater read depth. In order to identify the subset of strains that exhibited a significant change in splicing, we developed a statistical test that assessed the observed change in SI as a function of read depth (see *Materials and Methods*, Figure 2). Using this approach, statistically significant changes in SI were identified for 57 and 18 deletion strains for the *fet5*_intron1 and *pwi1*_intron2, respectively (see Figure 3). Importantly, of the 18 strains that affected the splicing of *pwi1*_intron2, 14 were also found to affect splicing of *fet5*_intron1. This significant degree of overlap (*P* < 3.81e-22, Fisher’s exact test) suggests that the splicing defect observed in many of the strains is not specific to a single gene.

To better understand the functional significance of the genes identified through this screen, we asked whether there was enrichment for factors involved in similar pathways by analyzing their Gene Ontology (GO) (The Gene Ontology Consortium 2000, 2015). Appropriately, the most highly enriched biological process identified was ‘mRNA splicing, via spliceosome’ (*P* < 6.63e-3, Table S3), confirming the ability of the method to positively identify known splicing factors. Consistent with our previous results in *S. cerevisiae*, not all deletions of known splicing factors resulted in a measurable change in splicing efficiency of either of the tested introns. Although these might represent false negative discoveries, on the basis of our experience in *S. cerevisiae*, we expect the more likely explanation is that these factors are not strictly required for efficient splicing of these specific introns under the conditions tested. Interestingly, significant overrepresentation of components of the SWR1 nucleosome remodeling complex was also uncovered (*P* < 6.75e-3), consistent with previous reports describing the role of SWR1 components in splicing (Patrick *et al.* 2015). Other GO categories that are well represented in the list of significant genes include ‘transcription from polymerase II promoter,’ ‘mRNA cleavage and polyadenylation specificity factor complex,’ and ‘chromatin remodeling’ (Figure 3 and Table S3).

Several of the genes identified here belong to seemingly unrelated GO categories. It seems important to reiterate that the approach implemented here does not measure defects in pre-mRNA splicing *per se*, but rather changes in the relative steady state levels of spliced and unspliced isoforms. As such, while some of these candidates may represent false positive discoveries, it seems likely that many are true positives, which impact splice isoform abundances either through nonsplicing related pathways or by modulating the activity of *bona fide* splicing factors. For example, deletions of either *ski2* or *trs130* resulted in some of the most significant increases in SI for either of the tested splicing events. Ski2 is an RNA helicase and member of the SKI complex, a highly conserved complex necessary for 3′ to 5′ degradation of transcripts subject to the nonsense-mediated decay (NMD) pathway (Mitchell and Tollervey 2003). The unspliced isoforms of *fet5* and *pwi1* contain premature stop codons and would be predicted targets of the NMD pathway, providing a plausible explanation for their accumulation in the Δ*ski2* strain. The *trs130* gene, in contrast, is involved in vesicle transport from the endoplasmic reticulum; the mechanism by which it might relate to altered splice isoform abundances is less clear. While no physical interactions have been documented between Trs130 and splicing-related proteins, epistatic genetic interactions between *trs130* and essential *bona-fide* splicing factors have been documented (Ryan *et al.* 2012).

To better understand the evolutionary nature of the genes that we identified, we examined each of them to determine whether homologs could be identified in either *S. cerevisiae* or humans. Of the 61 candidates we identified, 17 have clear human homologs but appear to lack an *S. cerevisiae* homolog (Table S4), underscoring the potential that *S. pombe* provides as a model system for understanding the complex splicing seen in mammalian systems. Four of these genes, *cay1*, *cwf18*, *cwf19*, and *pwi1*, have previously been annotated as splicing factors, while two others, *SPAC20H4.06c* and *SPBC713.05*, have been implicated in the splicing pathway on the basis of homology to human counterparts. Here, we provide experimental evidence that these protein products functionally impact pre-mRNA splicing.

### Known splicing factors identified here display global splicing defects

**Figure 4:**
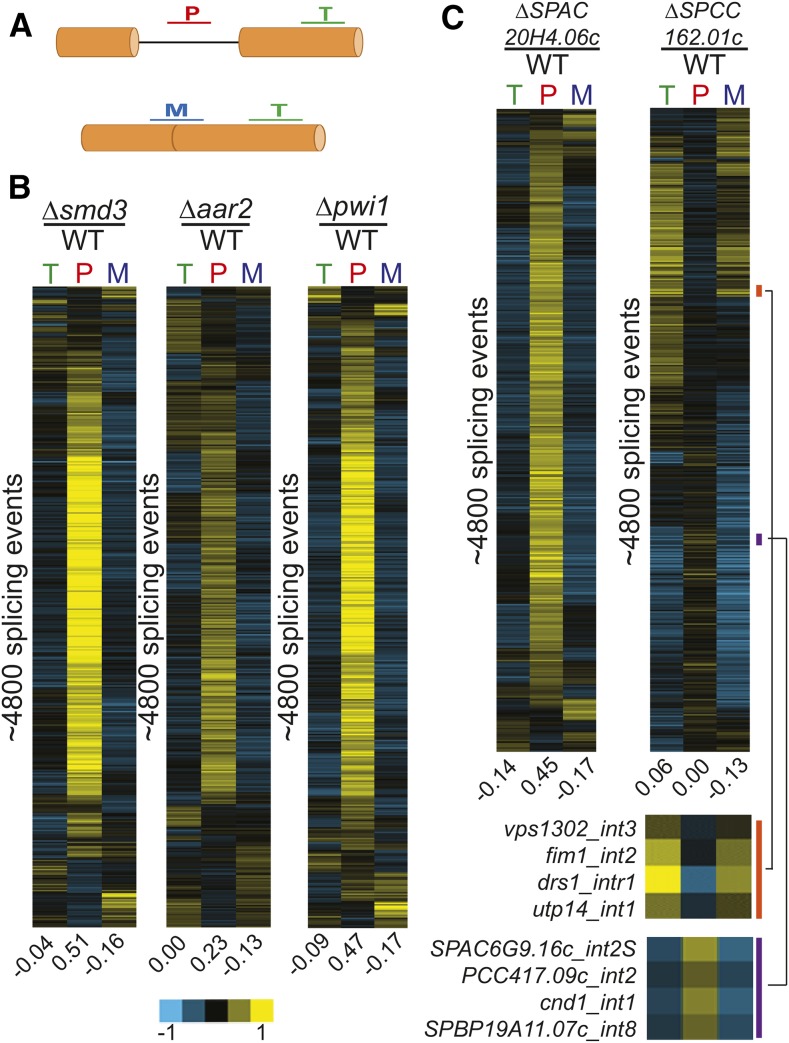
Known and predicted splicing factors display global splicing defects. (A) Splicing sensitive microarrays contain probes for quantification of total (T), pre-mRNA (P), and mature (M) mRNA levels. (B) Deletion of known splicing factors *smd3*, *aar2*, and *pwi1* each display global splicing defects. Each row represents the relative measurements for total, pre-mRNA, and mature mRNA for a particular splicing event. Numbers below each column represent the median value within the column. Rows from each sample are independently sorted by hierarchical clustering and displayed for only those events for which data were available for all three probe types. (C) Global splicing phenotypes of the Δ*SPAC20H4.06c* and Δ*SPCC162.01c* strains. The orange and purple bars highlight specific splicing events showing decreases or increases in splicing efficiency, respectively. mRNA, messenger RNA; WT, wild type.

To better understand the impact of the genes identified here, splicing sensitive microarrays were used to determine the global changes in pre-mRNA splicing that result from their deletions. These microarrays contain probes that target an exonic region of every protein coding gene in the *S. pombe* genome, as well as probes targeting every intron and its corresponding exon–exon junction, allowing for measurements of changes in total expression, pre-mRNA, and mature mRNA levels, respectively (Figure 4A). As an initial test, we chose to examine strains harboring deletions in three known splicing factors: (1) *smd3*, a core component of the SM complex in the U1, U2, U4, and U5 snRNPs; (2) *aar2*, a component of the U5 snRNP; and (3) *pwi1*, a splicing coactivator. For both *smd3* and *aar2*, clear homologs exist in both *S. cerevisiae* and humans, and their specific roles in the splicing pathways have been well characterized (Gottschalk *et al.* 2001; Nakazawa *et al.* 1991; Schwer and Shuman 2015). Moreover, the Δ*smd3* strain showed a statistically significant increase in our screen for both the *fet5*_intron1 and the *pwi1*_intron2 pre-mRNAs, while the Δ*aar2* strain showed increased pre-mRNA levels for both transcripts, albeit just below our cutoff for statistical significance. The third gene, *pwi1*, is the homolog of the human SRRM1 gene, a member of the SR-like family of proteins (Graveley 2000). Unlike *smd3* and *aar2*, there is no homolog of *pwi1* in the *S. cerevisiae* genome. In our screen data, deletion of *pwi1* caused a statistically significant increase in the SI of the *fet5*_intron1. Deletion of any of these three genes resulted in global defects in pre-mRNA splicing, albeit each with unique properties (Figure 4B). For each of the mutants, increased levels of pre-mRNAs were detected for a majority of the events observed, and concomitant decreases were seen for many of the mature mRNA species, consistent with our expectations for a *bona fide* splicing mutant. Furthermore, similar to our screen data, the Δ*aar2* strain showed levels of pre-mRNA accumulation that were overall lower than in the Δ*smd3* strain. Nevertheless, a similar number of splicing events was impaired by all three deletions.

### Predicted splicing factors also display global splicing defects

Among the genes we identified in our screen whose deletions negatively impacted the splicing of either *fet5*_intron1 or *pwi1*_intron2 were several that are predicted to be involved in the splicing pathway, based on homology studies. We chose to focus on two of these mutants, deletions of *SPAC20H4.06c*, an RNA-binding protein, and *SPCC162.01c*, a putative tri-snRNP component. Deletion of *SPAC20H4.06c* resulted in a statistically significant increase in both *fet5*_intron1 and *pwi1*_intron2 pre-mRNA levels, whereas deletion of *SPCC162.01c* also caused an increase in both pre-mRNAs, although just below our significance cutoff. As with *pwi1*, there are no apparent *S. cerevisiae* homologs for either *SPAC20H4.06c* or *SPCC162.01c*, but apparent human homologs do exist. For *SPAC20H4.06c*, the human homolog is GPATCH1, a member of the G-patch containing family of proteins. G-patch containing proteins have been previously implicated in splicing (Tsai *et al.* 2005), yet no direct evidence appears to exist that specifically couples GPATCH1 to splicing. In contrast, the human homolog of *SPCC162.01c* is SNRNP27, a component of the U4/U6·U5 tri-snRNP complex, and has a direct role in splicing (Fetzer *et al.* 1997). Interestingly, human SNRNP27 was previously shown to contain an N-terminal domain with strong homology to the SR domain of U170K; however, unlike U170K, SNRNP27 lacks an RNA-binding domain.

The global splicing defects of Δ*SPAC20H4.06c* and Δ*SPCC162.01c* revealed remarkably different phenotypes (Figure 4C). Deletion of *SPAC20H4.06c* showed a canonical splicing defect with broad increases in pre-mRNA species and decreases in mature mRNA species. The level to which pre-mRNAs accumulated is similar to that seen upon deletion of the canonical splicing factor *smd3* (Figure 4B). These data strongly suggest that the *SPAC20H4.06c* gene product is participating in the splicing pathway. In contrast, the global splicing profile resulting from deletion of *SPCC162.01c* looked quite different from the other splicing mutants examined here. Whereas a subset of splicing events appeared to be negatively affected by deletion of *SPCC162.01c*, as evidenced by the accumulation of pre-mRNA and loss of mature mRNA, a nearly equal number of splicing events seemed to be positively, albeit weakly, affected by its deletion, with pre-mRNA levels decreasing and mature mRNA levels increasing for these transcripts. These results are consistent with a model where SR proteins can function to either enhance or repress splice site activation at different introns. These data also suggest that a large number of *S. pombe* introns are spliced at suboptimal efficiency in wild-type cells. Additional experiments will be necessary to understand the mechanistic basis by which this SNRNP27 homolog can impart these phenotypes.

### Genes involved in heterochromatin formation show a range of genome-wide splicing defects

In examining the list of candidates identified in our screen, we were struck by the number of components with known roles involved in RNA silencing and heterochromatin formation. It has long been known that RNA plays a critical role in silencing in *S. pombe* via the RNA-induced initiation of transcriptional gene silencing (RITS) complex (Verdel *et al.* 2004). While it has been suggested that splicing components facilitate RITS function (Bayne *et al.* 2008), it remains unclear whether these effects are direct or indirect (Kallgren *et al.* 2014). Two groups recently described purifications of two related complexes involved in silencing: MTREC, which is involved in assembling heterochromatin (Lee *et al.* 2013); and the Nuclear RNA Silencing complex, NURS (Egan *et al.* 2014). While these complexes each contain unique elements, they share in common both the essential RNA helicase Mtl1 and the nonessential zinc-finger protein Red1. In our work, deletion of *red1* resulted in a statistically significant decrease in the splicing efficiency of both tested splicing events. In addition, affinity purification of Mtl1 as part of the MTREC complex copurified Ctr1 (Lee *et al.* 2013), whereas affinity purification of Red1 as part of the NURS complex copurified SPAC18G6.13 (Egan *et al.* 2014). In our screen, deletions of *ctr1* and *SPAC18G6.13* both resulted in statistically significant decreases in splicing efficiency of both target pre-mRNAs.

**Figure 5:**
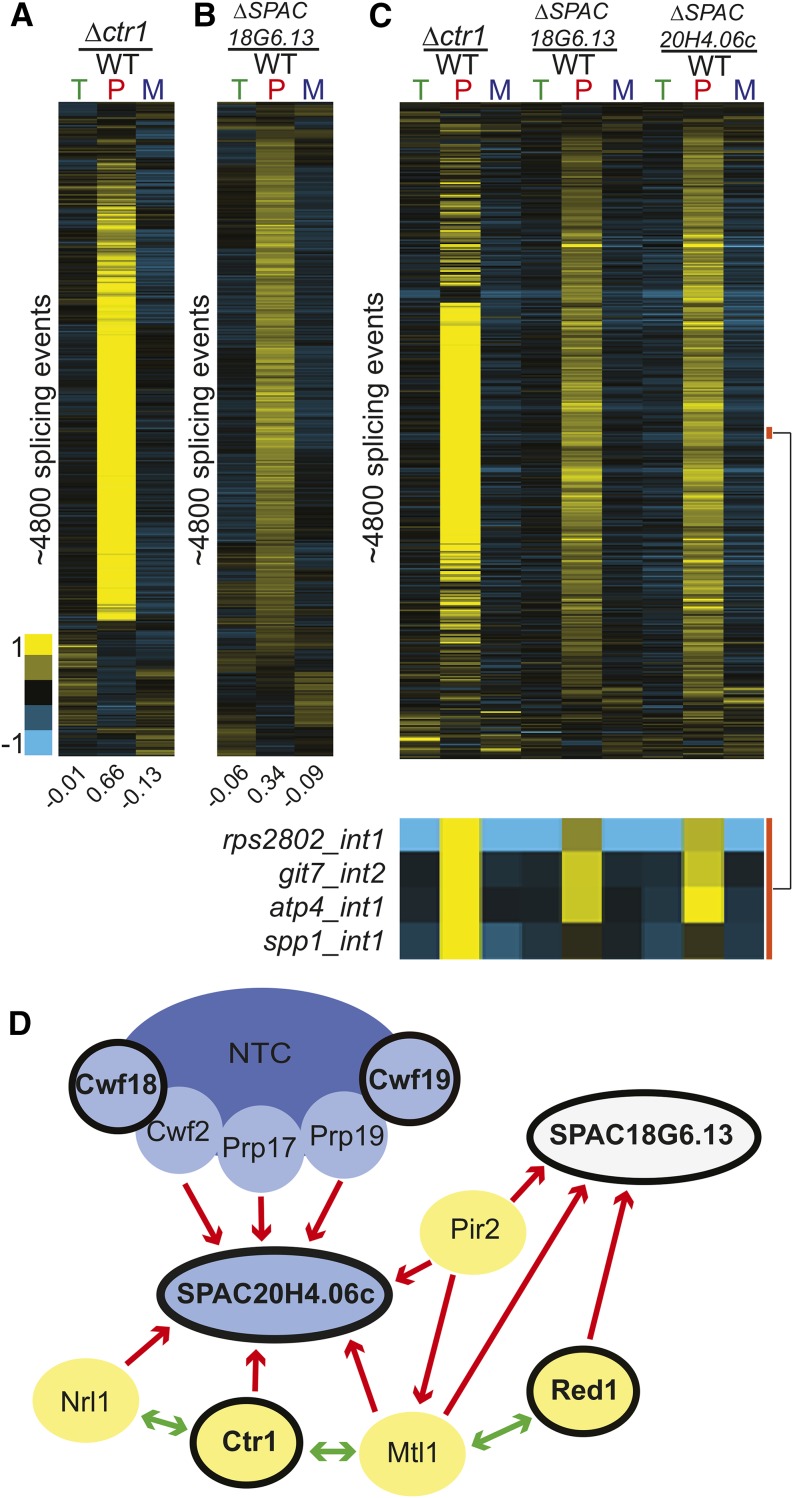
Deletion of factors involved in heterochromatin formation strongly impact global splicing. Splicing sensitive microarrays for Δ*ctr1* (A) and Δ*SPAC18G6.13* (B) reveal global splicing defects for each. Microarrays contain probes for quantification of total (T), pre-mRNA (P), and mature (M) mRNA levels. Splicing events for each mutant were sorted independently using hierarchical clustering and displayed for only those events for which data were available for all three probe types. (C) A comparison of the splicing defects on common targets reveals a large overlap among all three of these deletion strains, with a subset of events highlighted by the orange bar. (D) Known physical interactions between several components of the silencing pathway and the splicing pathway. Red arrows indicate previously published one-way physical interactions. Green arrows indicate two-way interaction. Blue ovals represent splicing factors, while yellow ovals represent members of the NURS and/or MTREC complexes. Black outlines note the components whose deletions caused splicing defects in this study. Previously described physical interactions between known splicing factors and components of the NURS and MTREC complexes, together with our observations that deletion of these components result in large accumulations of unspliced transcripts and decreases in spliced transcripts, suggest that these components may have a more direct role in splicing regulation. mRNA, messenger RNA; NURS, nuclear RNA silencing; WT, wild type.

Ctr1 was previously implicated in splicing of TER1, the RNA component of telomerase (Lee *et al.* 2013). Moreover, Ctr1 has been shown to physically interact with components of the Prp19 complex, including Cwf10, Cwf11, and Prp19. To determine whether Ctr1 had a more global effect on the splicing pathway, we again turned to microarray analysis. Deletion of *ctr1* resulted in a striking global splicing defect, strongly resembling that of a canonical splicing mutant (Figure 5A). A recent RNA-seq analysis of Δ*ctr1* and other MTREC mutants also revealed a global increase in unspliced transcript levels (Zhou *et al.* 2015). Interestingly, whereas our data reveal a broad decrease in mature mRNA concomitant with the increase in unspliced isoforms, the Zhou *et al.* (2015) study reported largely unchanged levels of spliced transcripts. Owing at least in part to this observation, Zhou and colleagues proposed that Ctr1/MTREC plays a role in targeting unspliced transcripts to the nuclear exosome, and that the pre-mRNA accumulation phenotype of the Δ*ctr1* strain did not reflect a direct role for MTREC on splicing. The broad decreases in mature mRNA demonstrated by our microarray experiments are more consistent with a direct role for Ctr1 in the splicing pathway; additional experiments will be necessary to understand the discrepancy between these results, and the functional significance of Ctr1 in the pre-mRNA splicing pathway.

In contrast to Ctr1, far less is known about the relationship between SPAC18G6.13 and splicing. Whereas physical interactions have been described between *SPAC18G6.13* and some splicing factors (Chen *et al.* 2014), the functional relevance of these interactions has not been previously described. Using microarray analysis, we showed that deletion of *SPAC18G6.13* also resulted in a broad increase in unspliced messages (Figure 5B). Interestingly, whereas SPAC18G6.13 was copurified with Red1 as part of the NURS complex, the same study also demonstrated that Mtl1 copurifies with SPAC20H4.06c, homolog of the human GPATCH1 gene described in the section above. When the global splicing defects of the Δ*ctr1*, Δ*SPAC18G6.13*, and Δ*SPAC20H4.06c* strains were analyzed together, the overlap in genome-wide splicing patterns was striking (Figure 5C). The physical interactions both between these proteins themselves and with additional components of the spliceosome as observed by others, as well as the splicing phenotypes we observed here in these mutants, suggest that the functional relationship between splicing and heterochromatin formation may be more bidirectional than previously thought (Figure 5D).

In addition to the NURS complex, heterochromatic silencing is accomplished in part through cooperation between the RNAi machinery and the heterochromatic factors Clr4 and the histone variant H2A.Z (Zofall *et al.* 2009; Grewal 2010; Hou *et al.* 2010; Anver *et al.* 2014). While H2A.Z is generally thought of as a repressive mark, it is also associated with promoters and may play roles in recruiting RNAP II to genes (Zlatanova and Thakar 2008). Interestingly, among our list of mutants that affected splicing of our targets were Δ*pht1*, the fission yeast homolog of H2A.Z, as well as many components of the INO80/SWR1complex, which is responsible for catalyzing H2A/H2A.Z exchange (Morrison and Shen 2009), including Δ*yaf9*, Δ*ies2*, Δ*vps71*, and Δ*swc2*. Similarly, while the Set1C complex is responsible for catalyzing the addition of H3K4me marks, it is also necessary for proper silencing of subtelomeric regions in *S. pombe* (Mikheyeva *et al.* 2014). Deletion of two components of the Set1complex, *ash2* and *swd1*, were identified in our screen as causing decreases in pre-mRNA splicing efficiency, although the Δ*swd1* effect was just below our significance cutoff (Table S1).

To determine the effect that loss of these heterochromatic factors have on the splicing pathway, we again assessed the global splicing profiles of the Δ*pht1*, Δ*ash2*, and Δ*swd1* strains by microarray. On the basis of these experiments alone, it is difficult to say whether deletion of any of these factors is impacting pre-mRNA splicing (Figure S3). While small groups of transcripts can be seen to exhibit a canonical splicing defect, the large changes in total gene expression, both increases and decreases, that are associated with these mutations complicates their analysis. Further studies will be necessary to better characterize the impact on pre-mRNA splicing of deletion of these genes.

### 3ʹ end processing factors impact the splicing of both terminal and nonterminal introns

**Figure 6:**
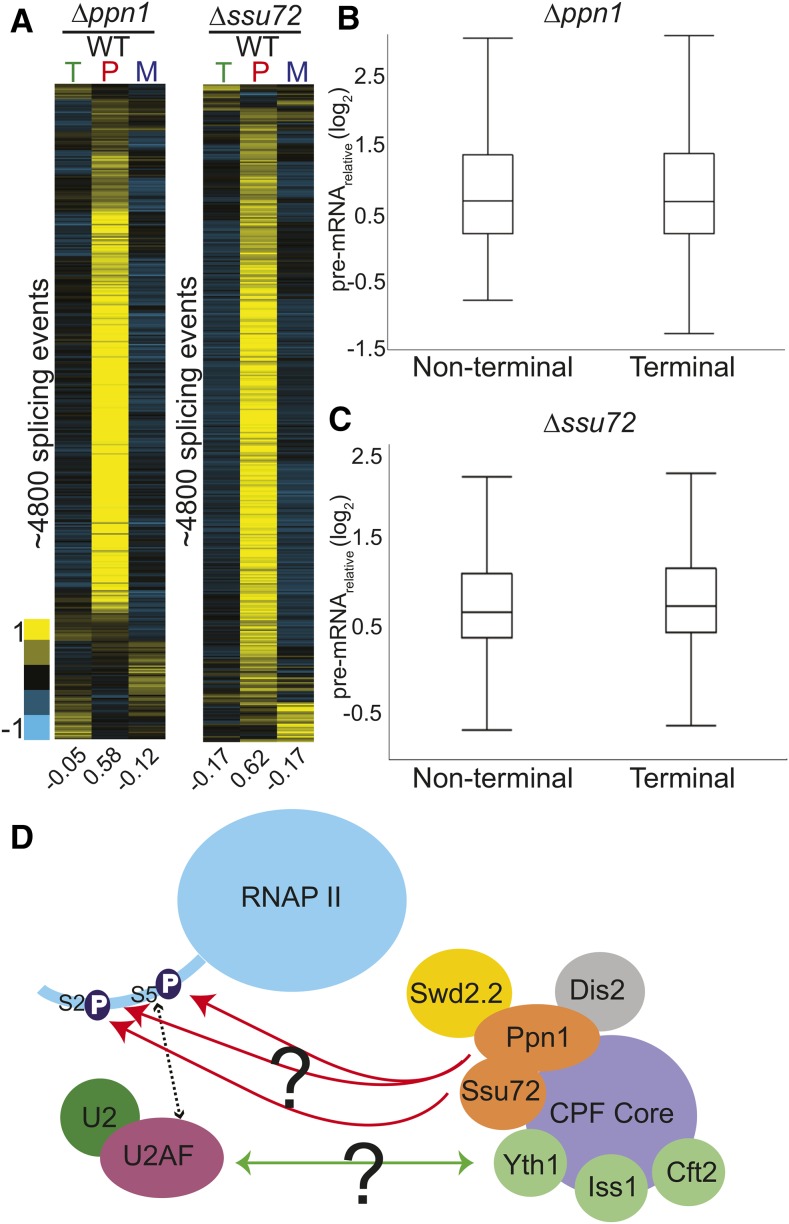
Deletions of 3ʹ end processing factors result in global splicing defects. Splicing sensitive microarrays for Δ*ppn1* (A) and Δ*ssu72* (B) strains each show broad splicing defects. Microarrays contain probes for quantification of total (T), pre-mRNA (P), and mature (M) mRNA levels. Splicing events from each array were clustered independently using hierarchical clustering and displayed for only those events for which data were available for all three probe types. (C) The pre-mRNA levels of terminal and nonterminal introns within multi-intronic genes were compared for each mutant, revealing no obvious difference between their behaviors. (D) Two potential mechanisms by which the CPF factors Ppn1 and Ssu72 may impact splicing are depicted: deletion of these factors could either prevent proper phosphorylation of the CTD tail and thus disrupt the interaction between U2AF and the CTD tail, or their absence from the CPF complex could disrupt physical interactions between splicing and cleavage and polyadenylation factors. Orange ovals represent CPF factors that cause significant splicing defects in our screen. Yellow ovals indicate factors that caused increases in pre-mRNA levels but were not deemed statistically significant. Green circles represent factors that have been shown in *S. cerevisiae* to cause splicing defects upon deletion. CPF, cleavage and polyadenylation factor complex; CTD, C-terminal domain; mRNA, messenger RNA; RNAP II, RNA polymerase II; WT, wild type.

Here, we identified two genes involved in the cleavage and polyadenylation pathway, *ssu72* and *ppn1*, whose deletions resulted in pre-mRNA splicing defects. The 3′ end processing and splicing pathways have been previously demonstrated to be functionally coupled together (Kyburz *et al.* 2006; Millevoi *et al.* 2006). Components of the U2 snRNP copurify with cleavage and polyadenylation specificity factor (CPSF), demonstrating a physical interaction between the two pre-mRNA processing pathways (Kyburz *et al.* 2006). In addition, CPSF is necessary for efficient splicing activity, while binding of the U2 snRNP promotes efficient cleavage at the 3′ end (Kyburz *et al.* 2006). Importantly, Ppn1 and Ssu72 have been shown to physically interact with each other in *S. pombe* and to copurify with the 3ʹ end processing machinery (Vanoosthuyse *et al.* 2014). We determined the global splicing profiles of these two mutants using microarrays: deletion of both *ssu72* and *ppn1* resulted in pre-mRNA splicing defects for a large fraction of the events monitored (Figure 6A). The defects seen for these mutants was similar to those seen for deletion of the canonical splicing mutant *smd3*, both in terms of the number of transcripts affected and the levels of pre-mRNA accumulation.

In higher eukaryotes, where exons are short and introns can be extraordinarily long, spliceosome assembly is hypothesized to occur by exon definition, wherein recognition of a downstream 5ʹ splice site can facilitate recognition of an upstream 3ʹ splice site by cross-exon interactions. For terminal introns, where no downstream 5ʹ splice site exists, it has been demonstrated that components of the cleavage and polyadenylation machinery can serve to facilitate recognition of the terminal 3ʹ splice site in a process termed terminal exon definition. Although it has been thought that the short introns in yeast would not require cross-exon interactions for efficient splicing, several studies have demonstrated that components of the cleavage and polyadenylation machinery do impact pre-mRNA splicing in yeast (Albulescu *et al.* 2012; Baejen *et al.* 2014). Given the large number of multi-intronic genes in *S. pombe*, we sought to determine whether the extent to which the pre-mRNA increases detected in these 3ʹ end mutants were dependent upon the locations of the intron. Each intron in the genome was classified as being either the last annotated intron (terminal) or not the last (nonterminal). The pre-mRNA increases we observed for terminal introns were not obviously different from the increases seen for nonterminal introns, neither in the Δ*ssu72* nor the Δ*ppn1* strains (Figure 6, B and C and Figure S4).

The mechanistic bases by which Ssu72 and Ppn1 influence pre-mRNA splicing remain unclear. Although they are physically parts of the CPSF complex, both Ssu72 and Ppn1 are phosphatases that target the CTD of RNA Pol II. Ssu72 preferentially targets the Ser5P modification (Rosado-Lugo and Hampsey 2014), while Ppn1 acts upon both Ser2P and Ser5P via the PP1 Nuclear Targeting Subunit (PNUTS) complex (Washington *et al.* 2002; Ciurciu *et al.* 2013). Phosphorylation of Ser5 is generally associated with promoter proximal pausing, and its dephosphorylation is important for escape into productive elongation (Rosado-Lugo and Hampsey 2014). The Ser5 mark of the CTD has been shown to be important for efficient splicing in yeast and humans, perhaps by slowing or pausing the polymerase so as to allow more time for cotranscriptional splicing to occur (Millhouse and Manley 2005; Alexander *et al.* 2010; Nojima *et al.* 2015). Given these roles for Ssu72 and Ppn1, the changes in splicing efficiency that accompany their deletions may not be a result of defects in cleavage and polyadenylation activity *per se*, but rather changes in the CTD phosphorylation state. Alternatively, our understanding of the interactions between the cleavage and polyadenylation machinery and splicing may be incomplete, such that the interactions known to be important for terminal exon definition may, in fact, be important for general spliceosome assembly. In budding yeast, where introns are strongly biased toward the 5′ end of transcripts, mutations in the endonuclease Brr5/Ysh1, ortholog of human CPSF-73, yield a strong splicing defect (Noble and Guthrie 1996), highlighting the capacity of *bona fide* 3′ end processing factors to influence splicing at distances far removed from locations of cleavage and polyadenylation. Moreover, affinity capture and mass-spectrometry analysis of the *S. pombe* cleavage and polyadenylation factor complex reveals physical interactions between PPN1 and both of the SR-protein orthologs in the *S. pombe* genome (Vanoosthuyse *et al.* 2014). More experiments will be necessary to understand the mechanistic bases by which 3ʹ end processing and splicing impact one another in *S. pombe*.

### Conclusions

Here, we described the development and implementation of a sequencing-based reverse genetic screen to identify the complement of nonessential genes in the fission yeast *S. pombe* that impact pre-mRNA splicing. Our ability to positively identify both known and predicted splicing factors demonstrates the ability of this approach to identify splicing mutants among a collection of thousands of diverse strains. Moreover, the identification here of scores of factors previously unknown to impact splicing highlights the potential of this approach for *de novo* discovery. As with all genetic screens, further characterization of the individual factors identified here will be necessary to understand the mechanistic bases by which each of them impacts the splicing pathway. Nevertheless, the broad interconnectivity of RNA-processing pathways revealed in this work is testimony to the utility of *S. pombe* as a genetic system for studying these processes. Moreover, the recently solved EM structure of the *S. pombe* spliceosome (Yan *et al.* 2015) significantly enhances the ability of genetic data to inform about the mechanistic underpinnings of this process. Importantly, because many known components of the spliceosome are themselves essential, their functions have not been assessed in the work described here. The creation of a collection of conditional alleles of essential genes and their subsequent analysis, using methods similar to those described here will present the opportunity to explore those essential genes, and thus provide greater insight into the mechanisms of more complex splicing.

## Supplementary Material

Supplemental Material
