# Supplementary material for: Interconnections Between RNA-Processing Pathways Revealed by a Sequencing-Based Genetic Screen for Pre-mRNA Splicing Mutants in Fission Yeast
Source: G3 (Bethesda). 2016 Mar 25;6(6):1513–23. doi: 10.1534/g3.116.027508 (PMC4889648; doi:10.1534/g3.116.027508)
Supplement: Supplemental Material [file supp_6_6_1513__index.html]

Interconnections Between RNA-Processing Pathways Revealed by a Sequencing-Based Genetic Screen for Pre-mRNA Splicing Mutants in Fission Yeast — Supplemental Material 

# Interconnections Between RNA-Processing Pathways Revealed by a Sequencing-Based Genetic Screen for Pre-mRNA Splicing Mutants in Fission Yeast

## Supplemental Material for Larson, Fair, and Pleiss, 2016

**Files in this Data Supplement:**

- Figure S1 - RT-PCR amplicon sequencing accurately and precisely measures splicing efficiency in complex mixtures containing known abundances of different spliced isoforms. (.pdf, 31 KB)
- Figure S2 - Splice index measurements for *fet5*\_intron1 and *pwi1*\_intron2 are reproducible. (.pdf, 170 KB)
- Figure S3 - Heterochromatin factors show splicing defects for a subset of splicing events. (.pdf, 92 KB)
- Figure S4 - 3' end processing factors affect the splicing of both terminal and not terminal introns. (.pdf, 176 KB)
- Table S4 - Many candidates identified in our screen have no apparent *S. cerevisiae* homolog but have an apparent homolog in humans. (.pdf, 51 KB)
- Table S1 - Read counts for spliced and unspliced isoforms and Splice Indices for both *fet5*\_intron1 and *pwi1*\_intron2 are shown for each of the strains examined. (.xlsx, 910 KB)
- Table S2 - Primer sequences used. (.xlsx, 10 KB)
- Table S3 - Results of GO analysis of identified strains. (.xlsx, 26 KB)
