# Supplementary material for: Interconnections Between RNA-Processing Pathways Revealed by a Sequencing-Based Genetic Screen for Pre-mRNA Splicing Mutants in Fission Yeast
Source: G3 (Bethesda). 2016 Mar 25;6(6):1513–23. doi: 10.1534/g3.116.027508 (PMC4889648; doi:10.1534/g3.116.027508)
Supplement: Supplemental Material [file supp_g3.116.027508_FigureS1.pdf]

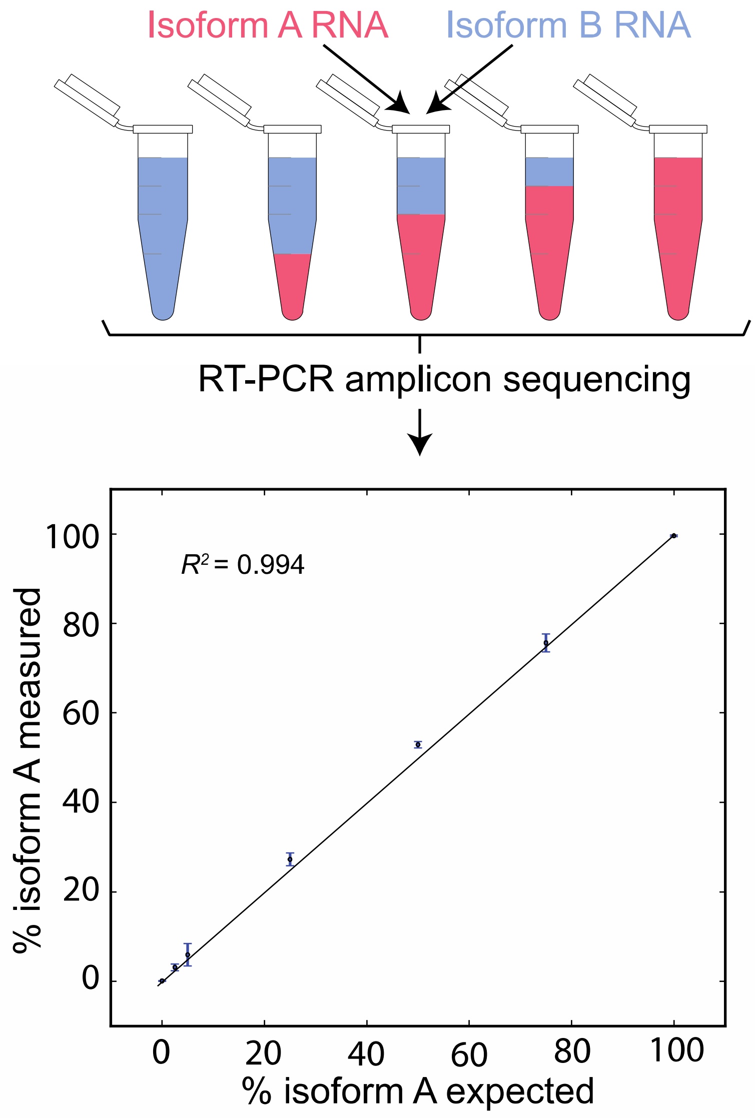

Figure S1: RT-PCR amplicon sequencing accurately and precisely measures splicing efficiency in complex mixtures containing known abundances of different spliced isoforms

Total cellular RNA was collected from two strains, each carrying a different splice isoform of the *pwi1* gene ORF in place of the native *pwi1* gene. Isoforms A and B differed by a length of 90 nucletotides. RNA from each strain was mixed in different proportions, and the relative abundance of each isoform was measured in each mixture by RT-PCR amplicon sequencing. Errorbars represent the standard deviation between three technical replicate RT-PCR reactions.
