# Supplementary material for: Interconnections Between RNA-Processing Pathways Revealed by a Sequencing-Based Genetic Screen for Pre-mRNA Splicing Mutants in Fission Yeast
Source: G3 (Bethesda). 2016 Mar 25;6(6):1513–23. doi: 10.1534/g3.116.027508 (PMC4889648; doi:10.1534/g3.116.027508)
Supplement: Supplemental Material [file supp_g3.116.027508_FigureS2.pdf]

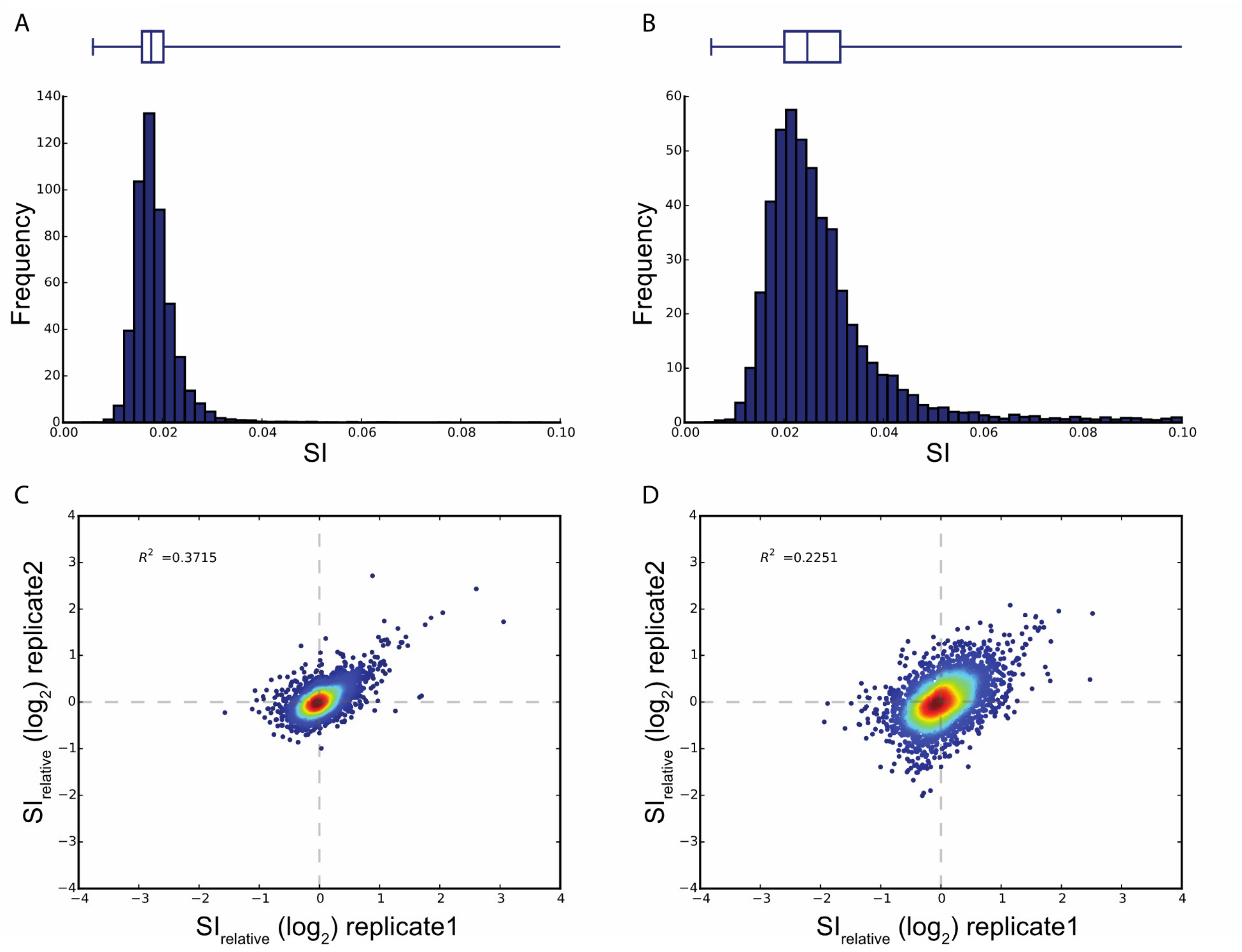

Figure S2: Splice index measurements for *fet5*_intron1 and *pwi1*_intron2 are reproducible

Histograms and box-whisker plots of the measured splice index for *fet5*_intron1 (A) and *pwi1*_intron2 (B) reveal that relatively consistent splice index measurements are generated for nearly all samples. The splice index relative to the median was calculated for each biological replicate for each deletion strain and replicate scatter plots (C, D) show that the relative splice index measurements are largely reproducible among the ~3000 strains in the collection.
