# Supplementary material for: Interconnections Between RNA-Processing Pathways Revealed by a Sequencing-Based Genetic Screen for Pre-mRNA Splicing Mutants in Fission Yeast
Source: G3 (Bethesda). 2016 Mar 25;6(6):1513–23. doi: 10.1534/g3.116.027508 (PMC4889648; doi:10.1534/g3.116.027508)
Supplement: Supplemental Material [file supp_g3.116.027508_FigureS3.pdf]

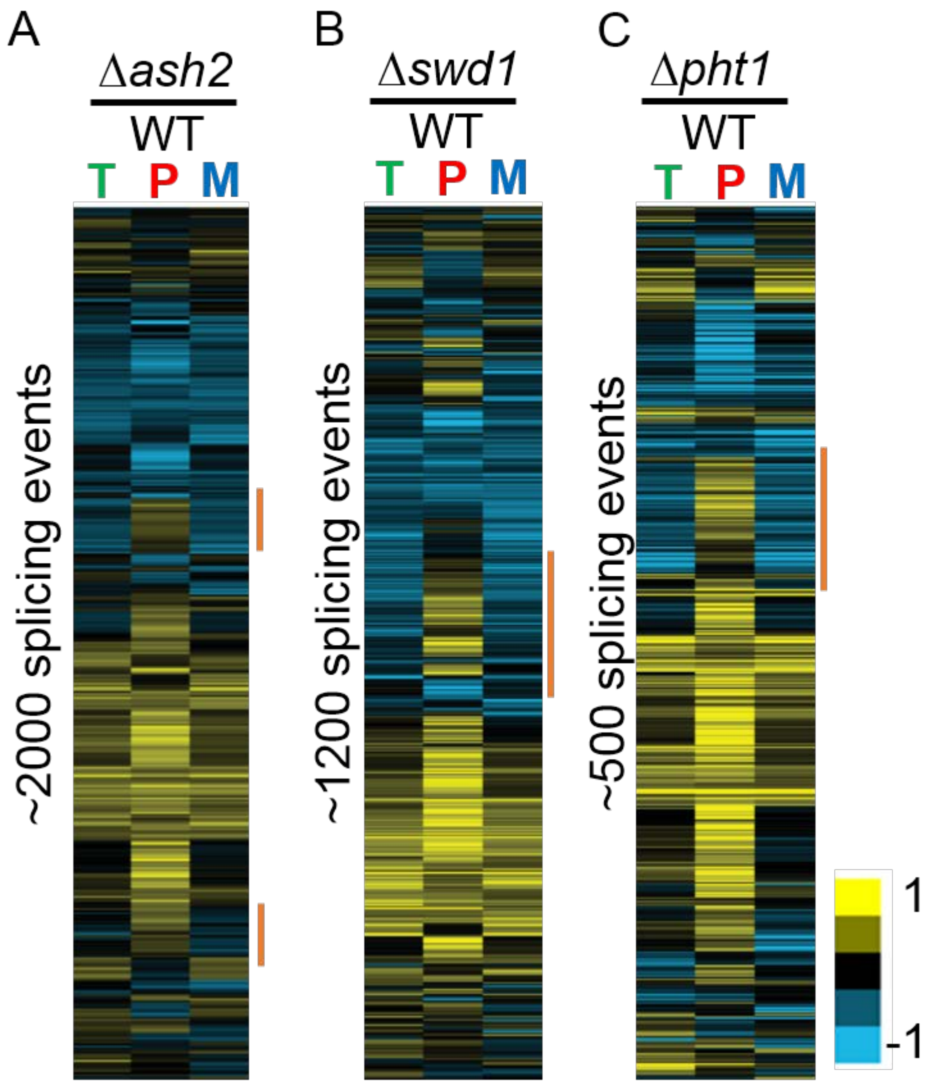

Figure S3: Heterochromatin factors show splicing defects for a subset of splicing events. Splicing sensitive microarrays for the heterochromatin factors *ash2* (A), *swd1* (B), and *pht1* (C). Each heatmap was independently organized using hierarchical clustering. The orange bar(s) in each panel indicate specific subsets of splicing events displaying canonical splicing defect phenotypes.
