# Supplementary material for: Interconnections Between RNA-Processing Pathways Revealed by a Sequencing-Based Genetic Screen for Pre-mRNA Splicing Mutants in Fission Yeast
Source: G3 (Bethesda). 2016 Mar 25;6(6):1513–23. doi: 10.1534/g3.116.027508 (PMC4889648; doi:10.1534/g3.116.027508)
Supplement: Supplemental Material [file supp_g3.116.027508_FigureS4.pdf]

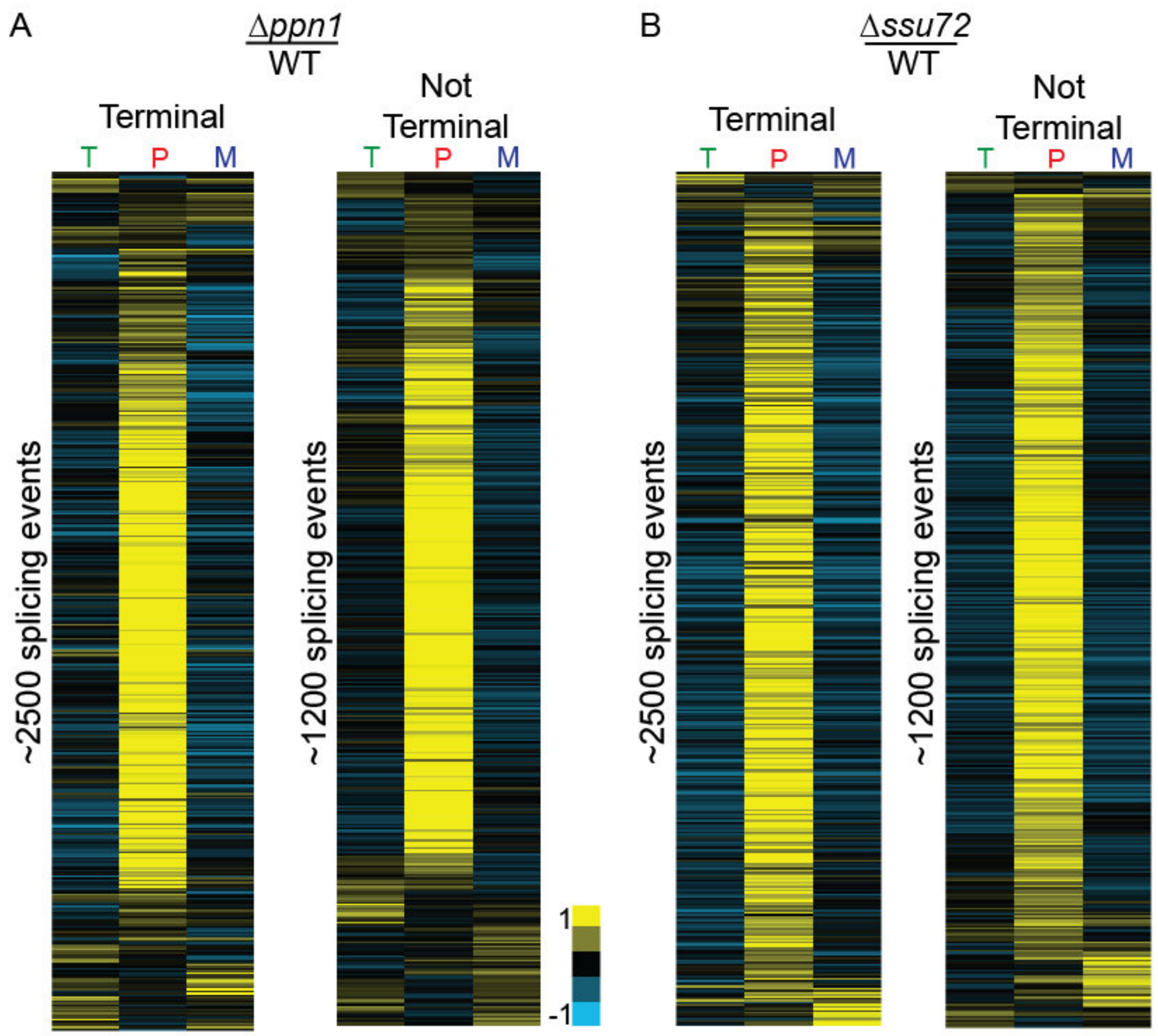

Figure S4: 3' end processing factors affect the splicing of both terminal and not terminal introns.

Splicing sensitive microarrays of 3' end processing factors *ppn1* (A) and *ssu72* (B). Only transcripts that contained multiple introns were considered for this analysis. Introns were called 'terminal' when they were the last intron annotated within a transcript, the remainder were considered 'not terminal'. Each heatmap was organized by hierarchical clustering. Global splicing defects are observed for each of the mutants, regardless of relative intron location.
