# Supplementary material for: Interconnections Between RNA-Processing Pathways Revealed by a Sequencing-Based Genetic Screen for Pre-mRNA Splicing Mutants in Fission Yeast
Source: G3 (Bethesda). 2016 Mar 25;6(6):1513–23. doi: 10.1534/g3.116.027508 (PMC4889648; doi:10.1534/g3.116.027508)
Supplement: Supplemental Material [file supp_g3.116.027508_TableS4.pdf]

	Candidate	*S. cerevisiae* homolog(s)	*H. sapiens* homolog
	ash2	*BRE2*	ASH2L
**	*cay1*	--	CACTIN
*	*csx2*	--	ACAP1
*	*ctr1*	--	CCDC174
**	*cwf18*	--	CCDC12
**	*cwf19*	--	CWF19L2/L1
	est1	*EST1*	SMG6
	hba2	*PDR5/10/12/15/18*	--
*	*hri2*	--	EIF2AK2/3
	ies2	*IES2*	INO80B
	imp1	*SRP1*	KPNA1/5/6
	lem2	*LEMD2*	SRC1, HEH2
	lsg1	*CTK3*	--
	med20	*SRB2*	MED20
	meu17	*SGA1*	--
	mug154	*NUR1*	--
	mug42	--	--
	mug78	*ATG13*	ATG13
	mug80	*CLG1*	--
	not3	*NOT3/5*	CNOT3
	nup170	*NUP157, NUP170*	NUP155
*	*nup37*	--	NUP37
	nxt3	*BRE5*	G3BP1
	oma4	*PMT4*	POMT1
	pds5	*PDS5*	PDS5B
	pht1	*HTZ1*	H2AFZ, H2AFV
	png2	*YNG1/2, PHO23*	ING2/3/4/5
	pof4	*EKA1*	TCEB3, TCEB3B
	ppk29	*PRK1, ARK1, AKL1*	BMP2K, AAK1
**	*ppn1*	--	PPP1R10
	prx	--	--
**	*pwi1*	--	SRRM1
*	*red1*	--	ZFC3H1
	rhp51	*RAD51*	RAD51
	rnh201	*RNH201*	RNASEH2A
	rpb9	*RPB9*	POLR21
	rpl2102	*RPL21A,B*	RPL21
	rpl4301	*RPL43A,B*	RPL37A

	Screen Hit	*S. cerevisiae* homolog(s)	*H. sapiens* homolog
*	*rtf2*	--	RTFDC1
*	*rtt109*	--	RTT109
*	*sdu1*	--	DES2
	smd3	*SMD3*	SNRPD3
	SPAC18G6.13	--	--
**	*SPAC20H4.06c*	--	GPATCH1
	SPAC22H10.11c	*IFH1, CRF1*	--
	SPBC1348.03	--	--
	SPBC28F2.08c	*HRD3*	SEL1L, SEL1L2, SEL1L3
*	*SPBC713.05*	--	WRD83
	SPCC18.13	*TRM82*	WDR4
	SPCC550.03c	*SKI2*	SKIV2L
	SPCC736.07c	*BUD27*	URI1
*	*sdj1*	--	PARK7
*	*spf31*	--	DNAJC8
	ssu72	*SSU72*	SSU72
	swc2	*VPS72*	VPS72
	swi3	*CSm3*	TIPIN
	toc1	--	--
	trs130	*TRS130*	TRAPPC10
	ubr1	*UBR1, UBR2*	UBR1, UBR2
	vps71	*VPS71*	ZNHIT1
	yaf9	*YAF9*	MLLT3, YEATS4

Table S4: Many candidates identified in our screen have no apparent *S. cerevisiae* homolog but have an apparent homolog in humans.

Listed here are the 61 candidates identified in our screen with their corresponding homolog(s) in *S. cerevisiae* and in humans. The red asterisks indicate the 17 genes that have no homolog in *S. cerevisiae* but do in humans. Of those 17 genes, the green asterisks indicate those genes that are known splicing factors. The blue asterisks indicate those genes that were predicted to be involved in splicing based on homology and have now been confirmed in this study.
